# Absorption, distribution and excretion of intravenously injected ^68^Ge/^68^Ga generator eluate in healthy rats, and estimation of human radiation dosimetry

**DOI:** 10.1186/s13550-015-0117-z

**Published:** 2015-07-17

**Authors:** Anu Autio, Helena Virtanen, Tuula Tolvanen, Heidi Liljenbäck, Vesa Oikonen, Tiina Saanijoki, Riikka Siitonen, Meeri Käkelä, Andrea Schüssele, Mika Teräs, Anne Roivainen

**Affiliations:** 1 Turku PET Centre, Turku University Hospital, University of Turku, FI-20521 Turku, Finland; 2 Turku Center for Disease Modeling, University of Turku, Turku, Finland; 3 Eckert & Ziegler Radiopharma GmbH, Berlin, Germany; 4 Preclinical Imaging and Drug Research, Turku PET Centre, University of Turku, Kiinamyllynkatu 4-8, FI-20521 Turku, Finland

**Keywords:** ^68^Ge/^68^Ga generator, Dosimetry, Rat, Whole-body distribution

## Abstract

**Background:**

This study evaluated the absorption, distribution, and excretion of Gallium-68 (^68^Ga) radionuclide after a single intravenous (i.v.) injection of ^68^Ge/^68^Ga generator eluate in healthy rats. Additionally, human radiation doses were estimated from the rat data.

**Methods:**

Twenty-one female and 21 male Sprague-Dawley rats were i.v. injected with 47 ± 4 MBq of ^68^Ge/^68^Ga generator eluate, and the radioactivity of excised organs was measured using a gamma counter at 5, 30, 60, 120, or 180 min afterwards (*n* = 3–7 for each time point). The radioactivity concentration and plasma pharmacokinetic parameters were calculated. Subsequently, the estimates for human radiation dosimetry were determined. Additionally, 4 female and 5 male rats were positron emission tomography (PET) imaged for *in vivo* visualization of biodistribution.

**Results:**

^68^Ga radioactivity was cleared relatively slowly from blood circulation and excreted into the urine, with some retention in the liver and spleen. Notably, the ^68^Ga radioactivity in female genital organs, i.e., the uterus and ovaries, was considerable higher compared with male genitals. Extrapolating from the female and male rat ^68^Ga data, the estimated effective dose was 0.0308 mSv/MBq for a 57-kg woman and 0.0191 mSv/MBq for a 70-kg man.

**Conclusions:**

The estimated human radiation burden of the ^68^Ge/^68^Ga generator eluate was slightly higher for females and similar for males as compared with somatostatin receptor ligands ^68^Ga-DOTANOC, ^68^Ga-DOTATOC, and ^68^Ga-DOTATATE, which is probably due to the retention in the liver and spleen. Our results revealed some differences between female and male rat data, which, at least in part, may be explained by the small sample size.

## Background

Gallium-68 (^68^Ga)-labeled tracers are increasingly used in positron emission tomography (PET) for diagnostic purposes. The prototypes of ^68^Ga-labeled PET imaging agents are the somatostatin receptor ligands, ^68^Ga-DOTATOC/NOC/TATE peptides, which are today routinely used for PET imaging of neuroendocrine tumors [1–3]. Currently, many other ^68^Ga-labeled peptide families are under clinical evaluation, such as bombesins (gastrin-releasing peptide receptor ligands), exendins (glucagon-like peptide 1 receptor ligands), and arginine-glycine-aspartic acids (RGDs) (integrin receptor ligands). In addition to oncology, ^68^Ga-tracer-based PET has been studied for imaging of inflammation [4].

^68^Ga offers a cyclotron-independent, convenient, and low-cost access to PET imaging agents. It is readily available by elution from a ^68^Germanium/^68^Gallium (^68^Ge/^68^Ga) generator possessing a 1-year life span depending on the uploaded ^68^Ge radioactivity. Furthermore, ^68^Ga has several convenient characteristics, such as β^+^ decay 89 %, Eβ^+^_max_ 1.9 MeV, and a sufficiently long half-life (67.71 min) for PET imaging.

The purpose of this study was to obtain preclinical information to support the use of the ^68^Ge/^68^Ga generator eluate for medical application. The absorption, distribution, and excretion of radioactivity after a single intravenous injection of the ^68^Ge/^68^Ga generator eluate were assessed in order to determine the radiation dosimetry of ^68^Ga. This study investigated the possible consequences of poor radiolabeling efficiency or *in vivo* dissociation of the radiolabeled conjugate, i.e., issues related to the effects produced in the patient by the free radionuclide. When ^68^Ga is eluted from ^68^Ge/^68^Ga generator with 0.1 mol/l hydrochloric acid solution, it is in the form of ^68^GaCl_3_. In aqueous solution; ^68^Ga is in the form of the hydrated gallium ion [Ga(H_2_O)_6_]^3+^. Insoluble neutral hydroxide colloids ^68^Ga(OH)_3_, may precipitated depending on pH (>4) and the concentration of ^68^Ga. After intravenous injection, the ^68^Ga radioactivity can migrate in the blood circulation as free ^68^Ga^3+^ or ^68^Ga^3+^ bound to transferrin, ferritin, or lactoferrin. Here, the absorption, distribution, and excretion of ^68^Ga radioactivity after a single intravenous (i.v.) injection were studied in healthy, mature Sprague-Dawley rats up to 3 h, and the estimates for human radiation dosimetry were calculated.

## Methods

All animal experiments were approved by the National Animal Experiment Board in Finland (ELLA) and the Regional State Administrative Agency for Southern Finland (ESAVI) and conducted in accordance with the relevant European Union Directive. This preclinical study was performed without randomization and blinding. The healthy, mature Sprague-Dawley rats were purchased from Harlan, The Netherlands, and they were of specific pathogen free (SPF) quality. The rats were left to acclimate for a minimum of 5 days after their arrival before the study. They were housed at room temperature (18–24 °C) and relative humidity of 40–70 %. Artificial lighting was used, with 12 h of light (6 a.m. to 6 p.m.) and 12 h of dark (6 p.m. to 6 a.m.). The animals received regular feed, and tap water was offered *ad libitum*. They fasted for 4–6 h prior to the administration of the ^68^Ge/^68^Ga generator eluate.

### ^68^Ge/^68^Ga generator eluate

The ^68^Ge/^68^Ga generator (Eckert-Ziegler Source No. 1484-7 with 1850 MBq nominal radioactivity at reference date) was eluted with 6 ml of 0.1 mol/l hydrochloric acid; the 0.7−1.2 ml radioactive elution peak was collected and diluted with phosphate-buffered saline (PBS) (600−860 μl, pH 7 ± 0) for i.v. injection.

### Biodistribution

Twenty-one female (weight 248 ± 13 g) and 21 male rats (weight 342 ± 47 g) were examined at five different time points post-injection (5, 30, 60, 120, and 180 min), with 3–7 female and 3–7 male rats per time point. The animals were placed in an immobilizer (AgnTho’s AB, Lidingö, Sweden), and a catheter was inserted in their tail vein. The rats were i.v. injected with 47 ± 4 MBq of ^68^Ge/^68^Ga generator eluate as a bolus and promptly flushed with physiological saline after injection. Animals were killed with an overdose of pentobarbital (Mebunat, Orion Pharma, Finland). Various organs were excised, weighed, and measured for total radioactivity by using a gamma counter (1480 Wizard 3" PerkinElmer/Wallac, Turku, Finland) cross-calibrated with the dose calibrator (VDC-404; Veenstra Instruments, Joure, The Netherlands). Of blood, bone, bone marrow, brown adipose tissue, fat, plasma, skeletal muscle, skin, and urine, only small samples were taken and measured for radioactivity and weight. All other tissues and organs were measured in their entirety. The residual carcass was assessed with a dose calibrator. Urine was obtained directly from the urinary bladder using a needle and syringe, and total radioactivity was measured as described above. Blood was obtained by means of cardiac puncture. Radioactivity of whole blood was measured. Plasma was separated by centrifugation (2118×*g* for 5 min at 4 °C), and plasma radioactivity was measured. The radioactivity concentration was decay corrected to the time of injection, and the results were expressed as standardized uptake values (SUV) and percentage of injected radioactivity dose per gram of tissue (%ID/g).

In addition to *ex vivo* studies, nine animals (five males 309–355 g, four females 245–287 g) were PET imaged for 180 min to visualize whole-body distribution* in vivo* and to obtain time-activity curves. The animals were anesthetized with isoflurane (induction 3 % and maintenance 1.7 %), and a catheter was inserted in their tail vein. The rats were i.v. injected with 45 ± 3 MBq of ^68^Ge/^68^Ga generator eluate and imaged by using a High Resolution Research Tomograph (HRRT; Siemens Medical Systems, Knoxville, TN, USA) PET camera. Both rats were imaged at the same time. They were kept on a warm pallet during the imaging procedure. For attenuation correction, a 6-min transmission scan was obtained using a collimated transmission point source. The PET imaging data were reconstructed using the ordered-subsets expectation maximization 3D algorithm (OSEM3D) with attenuation correction based on transmission source measurement.

### Plasma pharmacokinetics

Plasma pharmacokinetic parameters, i.e., the area under the curve (AUC), elimination rate constant (k_el_), total clearance (Cl_T_), and half-life (t_1/2_), were calculated using Microsoft Excel from the plasma concentrations at 5, 30, 60, 120, and 180 min after tracer injection. Only one sample could be obtained from each animal, wherefore the sampled concentrations from all animals (males and females separately) were combined in order to produce the curve of radioactivity concentration vs. time after injection. Since the injected radioactive dose per weight was not exactly the same in all animals, the plasma SUVs (radioactivity concentrations corrected by injected dose and animal weight) were used for calculation. Initial concentration (*C*_0_) was estimated by back-extrapolating from the log-linear regression of the two first concentration values (5- and 30-min samples). AUC between 0 and 180 min was calculated using the linear trapezoidal rule, starting from *C*_0_. Log-linear regression of the last three concentrations (60-, 120-, and 180-min samples) was used to estimate the AUC from 180 min to infinity that is, to calculate AUC_0–∞_ and to estimate k_el_ and t_1/2_. Because plasma concentrations were given in SUV units, the total clearance (CI_T_) is calculated as 1/AUC_0–∞_, and the unit of CI_T_ is then (g plasma/(g rat × min)).

### Estimation of human radiation dose

Absorbed doses were calculated with the OLINDA/EXM version 1.0 software (organ level internal dose assessment and exponential modeling; Vanderbilt University, Nashville, TN, USA), which applies the MIRD schema (developed by the Medical Internal Radiation Dose committee of the Society of Nuclear Medicine) for radiation dose calculations in internal exposure. The software includes radionuclide information and selection of human body phantoms. The residence times derived from the rat data were integrated as the area under the time-activity curve. The residence times were converted into corresponding human values by multiplication with a factor to scale the organ and body weights: (WTB_,rat_/W_Organ,rat_) × (W_Organ,human_/WTB_,human_), where WTB_,rat_ and WTB_,human_ are the body weights of rat and human (a 57-kg female or 70-kg male), respectively; and W_Organ,rat_ and W_Organ,human_ are the organ weights of rat and human (organ weights for a 57-kg female or 70-kg male), respectively.

### Statistical analysis

The mean values are calculated from the individual measurements and expressed with an accuracy of one standard deviation (mean ± SD). Differences between genders were assessed with Student’s *t* test.

## Results

**Fig. 1:**
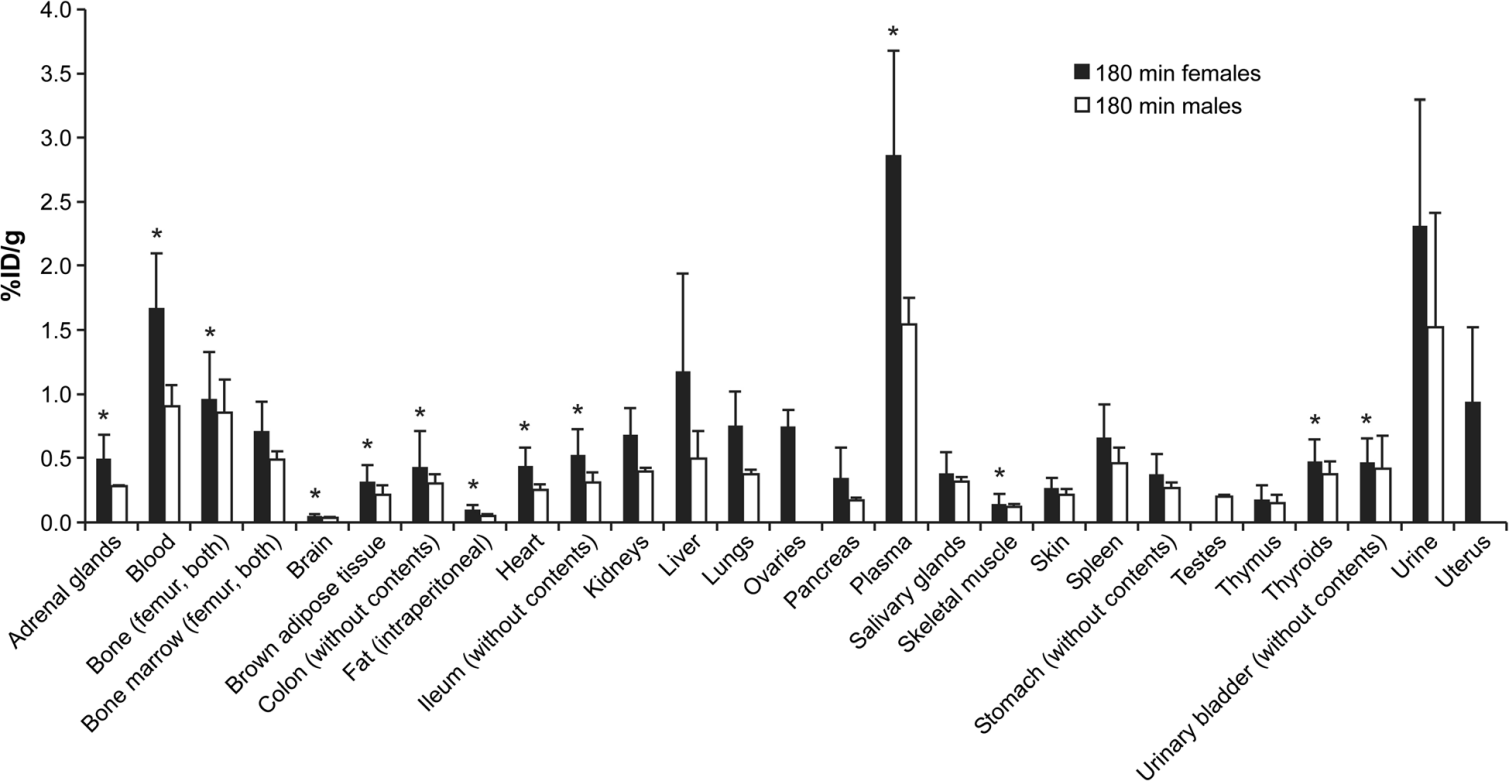
*Ex vivo* biodistribution of ^68^Ga radioactivity in rats at 180 min post-injection. Statistically significant (*P* < 0.05) differences between female and male are marked as *asterisk*

**Fig. 2:**
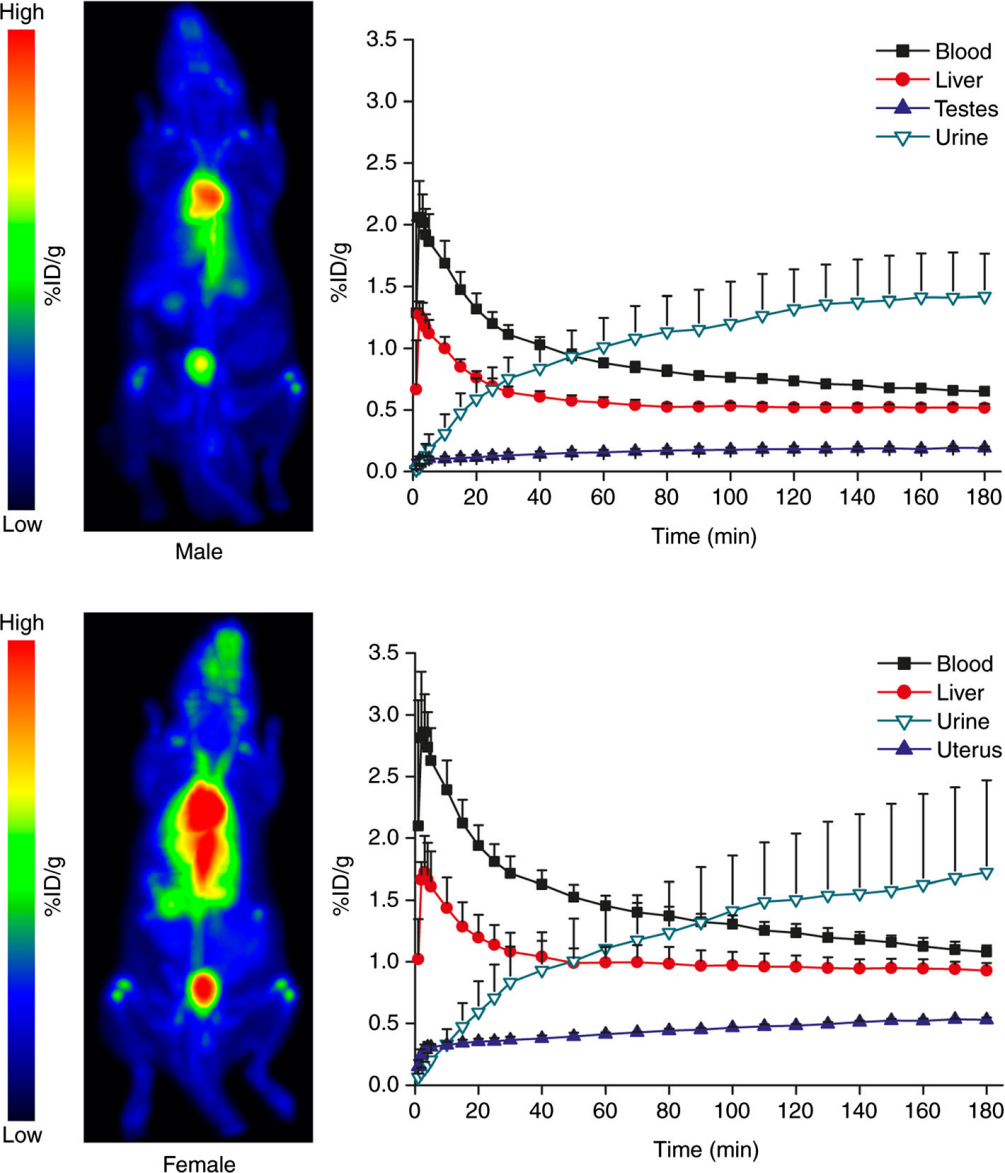
PET images and time-activity curves. *Left panels*: Healthy Sprague-Dawley rats were intravenously injected with ^68^Ge/^68^Ga generator eluate. Images are summations from 0–180 min after injection and displayed in the same color scale as percentage of injected dose per gram of tissue (%ID/g). Male rat: 350 g, 50 MBq (0.007 MBq/kg); female rat 259 g, 48 MBq (0.005 MBq/kg). *Right panels*: Mean time-activity curves of ^68^Ga radioactivity in selected organs/tissues of male (*upper*) and female (*lower*) rats. *Error bars* denote standard deviation. According to Student’s *t* test, the differences in blood (*P* = 0.00026) and testes vs. uterus (*P* < 0.0001) were statistically significant at 180-min post-injection. The “blood” values were obtained from heart left ventricle

Animal and tissue/organ weights and *ex vivo* biodistribution data from the rats are summarized in Tables 1 and 2, and 180-min results visualized in Fig. 1. ^68^Ga radioactivity was slowly cleared from blood circulation and excreted predominantly into the urine, with some retention in the liver and kidneys. Interestingly, the ^68^Ga radioactivity in female genital organs, i.e., the uterus and ovaries, was considerable higher than in male genitals (ovaries 0.750 %ID/g, uterus 0.941, testes 0.198 at 180 min). Plasma had also large difference 2.865 %ID/g (female) vs. 1.544 (male) at 180 min. *In vivo* PET images were in the line with *ex vivo* measurements (Fig. 2). Estimated plasma pharmacokinetic parameters for ^68^Ga radioactivity are given in Table 3. Plasma concentration was relatively high at the final time point (180 min), which may lead in uncertainty in the estimation of AUC_0–∞_.

**Table 1:** Organ weights and *ex vivo* biodistribution of ^68^Ga/^68^Ge generator eluate in female rats

Tissue/organ	Weight (g)	5 min	30 min	60 min	120 min	180 min
Adrenal glands	0.067 ± 0.016	0.673 ± 0.111	0.603 ± 0.271	0.705 ± 0.264	0.567 ± 0.226	0.497 ± 0.184
Blood	^a^	2.909 ± 0.272	2.509 ± 1.359	2.563 ± 0.909	2.128 ± 0.953	1.668 ± 0.428
Bone (femur, both)	^a^	0.298 ± 0.071	0.589 ± 0.132	0.820 ± 0.396	1.001 ± 0.363	0.963 ± 0.370
Bone marrow (femur, both)	^a^	0.586 ± 0.133	0.296 ± 0.051	0.705 ± 0.242	0.790 ± 0.366	0.710 ± 0.231
Brain	1.612 ± 0.103	0.075 ± 0.006	0.079 ± 0.040	0.084 ± 0.025	0.080 ± 0.041	0.052 ± 0.016
Brown adipose tissue	^a^	0.405 ± 0.017	0.605 ± 0.229	0.471 ± 0.213	0.376 ± 0.201	0.316 ± 0.126
Colon (without contents)	1.069 ± 0.288	0.289 ± 0.095	0.668 ± 0.283	0.615 ± 0.337	0.531 ± 0.302	0.431 ± 0.277
Fat (intraperitoneal)	^a^	0.058 ± 0.028	0.177 ± 0.093	0.145 ± 0.073	0.158 ± 0.151	0.099 ± 0.038
Heart	0.801 ± 0.049	0.723 ± 0.115	0.755 ± 0.462	0.617 ± 0.235	0.540 ± 0.204	0.439 ± 0.141
Ileum (without contents)	3.322 ± 1.420	0.271 ± 0.040	0.550 ± 0.191	0.465 ± 0.070	0.631 ± 0.426	0.520 ± 0.202
Kidneys	1.389 ± 0.162	0.773 ± 0.173	0.875 ± 0.328	0.823 ± 0.301	0.722 ± 0.324	0.679 ± 0.208
Liver	7.179 ± 0.836	1.225 ± 0.374	1.529 ± 0.450	1.490 ± 0.516	1.597 ± 0.627	1.178 ± 0.759
Lungs	1.117 ± 0.079	0.949 ± 0.212	1.078 ± 0.520	1.136 ± 0.379	0.894 ± 0.450	0.754 ± 0.268
Ovaries	0.135 ± 0.024	0.574 ± 0.174	0.784 ± 0.429	1.343 ± 1.257	0.782 ± 0.286	0.750 ± 0.124
Pancreas	1.180 ± 0.225	0.464 ± 0.283	0.389 ± 0.211	0.364 ± 0.174	0.331 ± 0.164	0.346 ± 0.233
Plasma	^a^	5.083 ± 0.486	4.491 ± 2.419	4.451 ± 1.523	3.742 ± 1.602	2.865 ± 0.813
Salivary glands	0.496 ± 0.047	0.397 ± 0.099	0.630 ± 0.230	0.506 ± 0.178	0.485 ± 0.229	0.382 ± 0.165
Skeletal muscle	^a^	0.132 ± 0.048	0.253 ± 0.080	0.214 ± 0.118	0.190 ± 0.078	0.143 ± 0.077
Skin	^a^	0.096 ± 0.029	0.341 ± 0.142	0.312 ± 0.176	0.265 ± 0.089	0.263 ± 0.079
Spleen	0.687 ± 0.136	0.493 ± 0.137	0.836 ± 0.204	0.785 ± 0.269	0.687 ± 0.201	0.661 ± 0.258
Stomach (without contents)	1.184 ± 0.216	0.261 ± 0.060	0.384 ± 0.117	0.471 ± 0.196	0.441 ± 0.255	0.369 ± 0.159
Thymus	0.305 ± 0.082	0.234 ± 0.003	0.259 ± 0.143	0.225 ± 0.089	0.264 ± 0.193	0.181 ± 0.106
Thyroids	0.017 ± 0.005	0.516 ± 0.020	0.656 ± 0.327	0.607 ± 0.245	0.576 ± 0.347	0.473 ± 0.174
Urinary bladder (without contents)	0.068 ± 0.021	0.256 ± 0.080	0.442 ± 0.172	0.636 ± 0.311	0.734 ± 0.187	0.469 ± 0.187
Urine	^a^	0.417 ± 0.134	13.162 ± 3.607	3.526 ± 1.783	5.228 ± 2.368	2.314 ± 0.981
Uterus	0.591 ± 0.153	0.361 ± 0.145	1.023 ± 0.482	1.149 ± 0.865	0.819 ± 0.553	0.941 ± 0.585
Residual carcass	208.418 ± 11.160	0.200 ± 0.010	0.318 ± 0.036	0.266 ± 0.025	0.269 ± 0.023	0.262 ± 0.029

Results are expressed as percentage of injected radioactivity dose per gram of tissue (mean ± SD)

^a^Of blood, bone, bone marrow, brown adipose tissue, fat, plasma, skeletal muscle, skin, and urine, only a small sample was taken and weighed. All other tissues/organs were measured in their entirety

**Table 2:** Tissue/organ weights and *ex vivo* biodistribution of ^68^Ga/^68^Ge generator eluate in male rats

Tissue/organ	Weight (g)	5 min	30 min	60 min	120 min	180 min
Adrenal glands	0.040 ± 0.012	0.635 ± 0.404	0.303 ± 0.047	0.338 ± 0.040	0.355 ± 0.174	0.278 ± 0.011
Blood	^a^	1.556 ± 0.476	1.119 ± 0.088	1.175 ± 0.128	0.970 ± 0.165	0.908 ± 0.157
Bone (femur, both)	^a^	0.285 ± 0.100	0.387 ± 0.188	0.500 ± 0.176	0.657 ± 0.240	0.854 ± 0.256
Bone marrow (femur, both)	^a^	0.494 ± 0.061	0.354 ± 0.020	0.423 ± 0.066	0.394 ± 0.123	0.490 ± 0.064
Brain	1.633 ± 0.163	0.078 ± 0.020	0.041 ± 0.003	0.048 ± 0.006	0.034 ± 0.002	0.035 ± 0.007
Brown adipose tissue	^a^	0.294 ± 0.099	0.279 ± 0.122	0.318 ± 0.075	0.255 ± 0.071	0.217 ± 0.070
Colon (without contents)	1.223 ± 0.424	0.230 ± 0.018	0.260 ± 0.048	0.285 ± 0.038	0.227 ± 0.077	0.305 ± 0.066
Fat (intraperitoneal)	^a^	0.050 ± 0.019	0.069 ± 0.013	0.090 ± 0.031	0.069 ± 0.046	0.053 ± 0.009
Heart	1.086 ± 0.113	0.494 ± 0.075	0.338 ± 0.070	0.320 ± 0.032	0.270 ± 0.053	0.251 ± 0.042
Ileum (without contents)	3.923 ± 1.768	0.205 ± 0.088	0.186 ± 0.022	0.268 ± 0.074	0.222 ± 0.114	0.308 ± 0.081
Kidneys	2.113 ± 0.279	0.993 ± 0.730	0.359 ± 0.070	0.404 ± 0.026	0.343 ± 0.119	0.397 ± 0.025
Liver	12.497 ± 1.444	1.044 ± 0.516	0.514 ± 0.195	0.770 ± 0.336	0.746 ± 0.421	0.498 ± 0.209
Lungs	1.277 ± 0.133	0.608 ± 0.028	0.479 ± 0.045	0.484 ± 0.196	0.455 ± 0.079	0.374 ± 0.037
Pancreas	1.307 ± 0.301	0.246 ± 0.042	0.207 ± 0.067	0.212 ± 0.031	0.181 ± 0.034	0.173 ± 0.016
Plasma	^a^	3.481 ± 0.980	2.078 ± 0.194	2.162 ± 0.251	1.639 ± 0.259	1.544 ± 0.203
Salivary glands	0.596 ± 0.060	0.351 ± 0.079	0.304 ± 0.018	0.336 ± 0.058	0.331 ± 0.075	0.315 ± 0.034
Skeletal muscle	^a^	0.085 ± 0.069	0.125 ± 0.036	0.142 ± 0.019	0.151 ± 0.028	0.123 ± 0.023
Skin	^a^	0.124 ± 0.074	0.157 ± 0.060	0.248 ± 0.038	0.204 ± 0.054	0.219 ± 0.037
Spleen	0.864 ± 0.135	0.520 ± 0.114	0.383 ± 0.099	0.516 ± 0.193	0.582 ± 0.255	0.455 ± 0.129
Stomach (without contents)	1.403 ± 0.241	0.222 ± 0.045	0.227 ± 0.061	0.268 ± 0.036	0.250 ± 0.058	0.264 ± 0.048
Testes	3.509 ± 0.214	0.076 ± 0.054	0.075 ± 0.023	0.140 ± 0.020	0.175 ± 0.026	0.198 ± 0.018
Thymus	0.497 ± 0.088	0.190 ± 0.033	0.137 ± 0.055	0.182 ± 0.050	0.141 ± 0.051	0.154 ± 0.064
Thyroids	0.020 ± 0.007	0.536 ± 0.040	0.355 ± 0.057	0.403 ± 0.131	0.415 ± 0.106	0.370 ± 0.105
Urinary bladder (without contents)	0.070 ± 0.015	0.267 ± 0.177	0.553 ± 0.360	0.502 ± 0.191	0.478 ± 0.216	0.418 ± 0.259
Urine	^a^	0.758 ± 0.727	1.342 ± 0.530	1.408 ± 0.736	3.111 ± 3.927	1.520 ± 0.894
Residual carcass	289.423 ± 45.504	0.177 ± 0.082	0.218 ± 0.051	0.207 ± 0.039	0.215 ± 0.048	0.206 ± 0.038

Results are expressed as percentage of injected radioactivity dose per gram of tissue (mean ± SD)

^a^Of blood, bone, bone marrow, brown adipose tissue, fat, plasma, skeletal muscle, skin, and urine, only a small sample was taken and weighed. All other tissues/organs were measured in their entirety

**Table 3:** Plasma pharmacokinetic parameters for ^68^Ga radioactivity after an intravenous bolus injection of ^68^Ga/^68^Ge generator eluate in rats

Parameter	Males (*n* = 21)	Females (*n* = 21)
k_el_ (1/h)	0.175349	0.201682
t_1/2_ (h)	3.952956	3.436839
AUC_0–∞_ (h × g/g)	48.66309	65.64807
Cl_T_ (g plasma/(g rat × h))	0.020549	0.015233

*AUC* area under the curve, *k*_*el*_ elimination rate constant, *Cl*_*T*_ total clearance, *C*_*0*_ initial concentration, *t*_*1/2*_ plasma half-life

The human residence times for the various source organs and radiation dose estimates for ^68^Ga radioactivity, extrapolated from rat biodistribution data, are listed in Table 4. The estimations of the absorbed doses were calculated for a 70-kg adult male and a 57-kg adult female. Extrapolating from the female rat data, the effective dose for a 57-kg adult female was 0.0308 mSv/MBq, i.e., 7.7 mSv from an intravenously injected radioactivity of 250 MBq. The corresponding estimates for 15-, 10-, 5-, and 1-year-old and newborn females are presented in Table 5. Estimated from female rat data, the absorbed doses were the greatest in heart wall (0.501 mSv/MBq), osteogenic cells (0.076 mSv/MBq), liver (0.072 mSv/MBq), lungs (0.0502 mSv/MBq), and spleen (0.034 mSv/MBq). Extrapolating from the male rat data, the effective dose for a 70-kg adult male was 0.0191 mSv/MBq, i.e., 4.8 mSv from an intravenously injected radioactivity of 250 MBq. The corresponding estimates for 15-, 10-, 5-, and 1-year-old and newborn males are presented in Table 6. Estimated from the male rat data, the absorbed doses were greatest in heart wall (0.216 mSv/MBq), liver (0.0652 mSv/MBq), osteogenic cells (0.0418 mSv/MBq), urinary bladder wall (0.0382 mSv/MBq), and lungs (0.0245 mSv/MBq).

**Table 4:** Human residence times and radiation dose estimates for ^68^Ga radioactivity extrapolated from the rat biodistribution data

Organ	Residence time (h)		Dose (mSv/MBq)	
	Female rat data	Male rat data	Female rat data	Male rat data
Adrenals			0.02210	0.01600
Blood	0.7790	0.3708		
Bone	0.3120	0.1950		
Bone marrow	0.0688	0.0425		
Brain	0.0064	0.0043	0.00591	0.00372
Breasts			0.01790	0.01260
Gallbladder wall			0.01950	0.01680
Lower large intestine wall			0.01520	0.01260
Small intestine			0.01480	0.01350
Stomach wall			0.01870	0.01400
Upper large intestine wall			0.01580	0.01360
Heart wall	0.0117	0.0072	0.50100	0.21600
Kidneys	0.0149	0.0118	0.03360	0.02430
Liver	0.1716	0.2226	0.07220	0.06520
Lungs	0.0591	0.0352	0.05020	0.02450
Muscle	0.4238	0.2484	0.01900	0.00928
Ovaries	0.0006		0.03080	0.01290
Pancreas	0.0027	0.0013	0.02860	0.01530
Red marrow	0.0688	0.0425	0.02530	0.01850
Osteogenic cells			0.07580	0.04180
Skin			0.01170	0.0097300
Spleen	0.0081	0.0069	0.03370	0.02280
Thymus			0.03050	0.01860
Thyroid	0.0008	0.0005	0.02640	0.02100
Testes		0.0005		0.00934
Urinary bladder wall			0.01350	0.03820
Uterus	0.0006		0.01140	0.01320
Whole body			0.02560	0.01730
Effective dose			0.0308	0.0191

**Table 5:** Additional dosimetry estimates based on a female rat distribution data

	Absorbed dose per unit radioactivity administered (mSv/MBq)				
Organ	15-year-olds	10-year-olds	5-year-olds	1-year-olds	Newborn
	(50 kg)	(30 kg)	(17 kg)	(10 kg)	(5 kg)
Adrenals	0.0220	0.0337	0.0521	0.0922	0.212
Brain	0.00526	0.00685	0.00531	0.0148	0.0333
Breasts	0.0178	0.0304	0.0483	0.0885	0.211
Gallbladder wall	0.0194	0.0307	0.0466	0.0875	0.217
Lower large intestine wall	0.0144	0.0236	0.0374	0.0696	0.176
Small intestine	0.0157	0.0251	0.0400	0.0762	0.182
Stomach wall	0.0187	0.0285	0.0451	0.0849	0.202
Upper large intestine wall	0.0153	0.0247	0.0400	0.0754	0.185
Heart wall	0.576	0.893	1.44	2.61	5.11
Kidneys	0.0363	0.0521	0.0777	0.139	0.351
Liver	0.0722	0.109	0.164	0.315	0.721
Lungs	0.0575	0.0823	0.125	0.239	0.611
Muscle	0.0201	0.0382	0.109	0.215	0.305
Ovaries	0.0349	0.0893	0.1550	0.349	0.710
Pancreas	0.0335	0.0608	0.0823	0.166	0.496
Red marrow	0.0262	0.0455	0.0828	0.201	0.737
Osteogenic cells	0.0745	0.117	0.194	0.452	1.39
Skin	0.0116	0.0187	0.0306	0.0595	0.151
Spleen	0.0391	0.0602	0.0955	0.174	0.449
Testes	0.0126	0.0203	0.0327	0.0637	0.156
Thymus	0.0311	0.0413	0.0622	0.107	0.237
Thyroid	0.0342	0.0537	0.113	0.213	0.314
Urinary bladder wall	0.0733	0.113	0.180	0.345	0.895
Uterus	0.0118	0.0721	0.110	0.198	0.136
Total body	0.0258	0.0420	0.0678	0.133	0.338
Effective dose (mSv/MBq)	0.03110	0.0582	0.096800	0.20100	0.48500

**Table 6:** Additional dosimetry estimates based on a male rat distribution data

	Absorbed dose per unit radioactivity administered (mSv/MBq)				
Organ	15-year-olds	10-year-olds	5-year-olds	1-year-olds	Newborn
	(50 kg)	(30 kg)	(17 kg)	(10 kg)	(5 kg)
Adrenals	0.0201	0.0313	0.0491	0.0897	0.231
Brain	0.0039	0.00523	0.00723	0.0115	0.0259
Breasts	0.01580	0.0258	0.0416	0.0790	0.195
Gallbladder wall	0.0203	0.0312	0.0483	0.0921	0.228
Lower large intestine wall	0.00149	0.0248	0.0396	0.0746	0.191
Small intestine	0.0167	0.0267	0.0427	0.0818	0.199
Stomach wall	0.0175	0.0275	0.0440	0.0843	0.206
Upper large intestine wall	0.0165	0.0266	0.0428	0.0814	0.202
Heart wall	0.280	0.435	0.700	1.27	2.49
Kidneys	0.0295	0.0421	0.0633	0.113	0.282
Liver	0.0876	0.133	0.199	0.384	0.888
Lungs	0.0352	0.0504	0.0765	0.147	0.374
Muscle	0.0137	0.0253	0.0665	0.134	0.199
Ovaries	0.0175	0.0258	0.0461	0.0799	0.194
Pancreas	0.02060	0.0361	0.0501	0.098	0.275
Red marrow	0.02190	0.0355	0.0643	0.1620	0.630
Osteogenic cells	0.05470	0.0850	0.1390	0.3220	0.974
Skin	0.0121	0.0198	0.0326	0.0639	0.164
Spleen	0.0327	0.0503	0.0801	0.1460	0.378
Testes	0.0185	0.1120	0.1320	0.1810	0.269
Thymus	0.02270	0.0324	0.0501	0.0911	0.214
Thyroid	0.0330	0.0514	0.11	0.206	0.298
Urinary bladder wall	0.0488	0.0760	0.122	0.232	0.6040
Uterus	0.0165	0.0262	0.0424	0.0810	0.197
Total body	0.0220	0.0360	0.0584	0.1150	0.292
Effective dose (mSv/MBq)	0.02530	0.05580	0.081000	0.14600	0.34300

## Discussion

The latest research has produced a series of new compounds labeled with ^68^Ga, which is a positron-emitting metal especially suitable for labeling of peptides and PET. In this study, we investigated the absorption, distribution, and excretion of ^68^Ga/^68^Ge radioactivity after a single intravenous injection of ^68^Ge/^68^Ga generator eluate in female and male rats over a period of 3 h.

The observed differences between the female and male biodistribution data are probably, at least in part, due to the small number of animals. However, higher concentrations of some elements, including iron, have been observed in the organs of female rats than in male rats, and since Ga^3+^ is transported into tissues in a similar way as Fe^3+^, this may explain part of the gender difference observed in this study [5]. This, in addition to higher injected radioactivity dose per gram, might in part explain higher plasma values of female rats. Interestingly, the estimated human radiation dose was higher when using the female rat data than when using the male rat data. Possible explanations for this include the small sample size and/or actual gender dependent differences in handling certain elements [5]. The body weight of female rats was approximately 100 g lower than that of male rats. However, both genders received an identical 50-MBq dose of ^68^Ga eluate. This may also be one source of difference.

When the studies started, the ^68^Ge/^68^Ga generator was already used for 7 month; the studies lasted up to 5 month. According to manufacturer’s metals screen by inductively coupled plasma mass spectrometry (ICP-MS), the ^68^Ge/^68^Ga generator (no. 1484-7, with 1850-MBq nominal radioactivity) had antimony 0.004 ppm, boron 0.17 ppm, sodium 0.56 ppm, titanium 0.069 ppm, and zinc 0.006 ppm, which reflects the typical metal impurities in the eluate. In the respective Ph. Eur. monograph, only zinc and iron are mentioned as metal impurities, because they might interfere as 3+ metals with the Ga-68 during the labeling. The limit of zinc and iron is 10 μg/GBq. The generator has a zinc level of 0.02 μg/GBq, which is well below the limit, and iron was not detected by ICP-MS by a detection limit of 0.006 ppm, which also correlates to a value way below the limit. The ^68^Ge breakthrough was 0.000017 % correlated to the ^68^Ga radioactivity of the eluate at the reference date. This value is well below the limit of ^68^Ge-breakthrough mentioned in the respective Ph. Eur. monograph. Thus, it was estimated that these levels of metal impurities and ^68^Ge breakthrough had no effect on the results. However, the chemical form of ^68^Ga used in this study (^68^Ga eluate was mixed with a phosphate-buffered saline) might be different from ^68^Ga-species present as by-products in ^68^Ga-labeled radiopharmaceuticals. In radiolabeling peptides, ^68^Ga is reacted with chelate-conjugated peptides at elevated temperature, which should accelerate the hydrolysis reaction of Ga that does not form complex with peptide-chelate conjugate.

The whole-body distribution of ^68^Ga radioactivity reported here is in line with previous publications. Velikyan and co-workers studied biodistribution of ^68^GaCl_3_ in healthy Sprague-Dawley rats in order to control the organs where the accumulation would occur in case of impure tracer or* in vivo* release of ^68^Ga from the tracer (^68^Ga-DOTATOC and ^68^Ga-DOTATATE). The ^68^GaCl_3_ was acetate buffered to pH of 4.6 and formulated with phosphate-buffered saline (pH 7.4) for i.v. injection. The ^68^Ga radioactivity concentration at 75-min post-injection was the highest in the blood, and the accumulation in the heart, lung, liver, and spleen was considerably higher as compared to that of peptide tracers [6]. Previously, we i.v. injected NaOH neutralized ^68^Ga-chloride (12 MBq from Cyclotron Co. ^68^Ge/^68^Ga generator, Obninsk, Russia) in anesthetized, athymic, male Hsd/RH-rnu/rnu rats having subcutaneous tumor xenografts and reported the following SUVs at 90 min after injection (the values at 120 min of the present report are given in the parentheses): blood 2.7 ± 0.3 (3.074 ± 0.337), liver 5.9 ± 3.3 (2.280 ± 0.918), lung 1.9 ± 0.8 (1.445 ± 0.195), muscle 0.2 ± 0.03 (0.478 ± 0.067), and skin 0.5 ± 0.1 (0.662 ± 0.233) [7]. Subsequently, we also studied distribution of ^68^GaCl_3_ (Cyclotron Co., Obninsk, Russia) in healthy C57BL/6 N mice. The ^68^GaCl_3_ was neutralized with 1 mol/l sodium hydroxide to pH of 7, and the final product contained 13 % of colloidal forms of ^68^Ga as determined by ultrafiltration. Still, the biodistribution of ^68^Ga radioactivity was quite similar to the present study. The highest level of ^68^Ga radioactivity at 3-h post-injection was found in the blood and liver followed by spleen, kidneys, bone with bone marrow, and lung, respectively [8]. Nanni and co-workers have studied ^68^Ga-citrate in patients with infectious diseases [9]. Since citrate is only a weak chelator of ^68^Ga, the radionuclide is rapidly released *in vivo *and subsequently binds to transferrin and some other plasma proteins. The biodistribution of ^68^Ga-citrate may actually resemble that of free ^68^Ga or the eluate of the ^68^Ge/^68^Ga generator. In clinical whole-body PET scanning, ^68^Ga-citrate showed relatively high vascular radioactivity, moderate hepatic uptake, mild bone marrow radioactivity, and no bowel radioactivity. The relatively high vascular radioactivity, which is not seen in ^67^Ga-citrate scintigraphy, was a particularly interesting finding. In the current rat study, the elimination of radioactivity in urine and feces at each time point and the overall mass balance as a percentage of administered radioactivities could not be determined since urine or feces were sampled, not collected in their entirety. The observed high plasma values in rats supports that the ^68^Ga radioactivity is bound to transferrin.

^68^Ga has been used extensively for the labeling of synthetic peptides. However, there are only few human dosimetry reports available, including, for example, those on peptide analogues that bind to somatostatin receptors (Table 7) [10–15]. The effective dose of the ^68^Ge/^68^Ga generator eluate reported here is somewhat higher than that of ^68^Ga-DOTANOC and ^68^Ga-DOTATOC [10, 11]. The higher dose of the ^68^Ge/^68^Ga generator eluate can be explained by the slow clearance from the blood and the retention in the liver. The biodistribution of ^68^Ga-labeled complexes is determined by the pharmacokinetics of the complexing molecules, such as peptides, and not by the incorporated Ga^3+^.

**Table 7:** Human effective doses of ^68^Ga radiopharmaceuticals and ^18^ F-FDG

Radiopharmaceutical	Effective dose (mSv/MBq)	Reference
^68^Ga-DOTANOC	0.025	Pettinato 2008 [10]
^68^Ga-DOTATOC	0.023	Hartmann 2009 [11]
^68^Ga-DOTATATE	0.021	Sandström 2013 [12]
^68^Ga-NOTA-2Rs15d	0.0218^a^	Xavier 2013 [13]
BAY86-7548	0.051	Roivainen 2013 [14]
^18^F-FDG	0.0190	ICRP Publication 1998 [15]
^68^Ga-eluate	0.0308^b^, 0.0191^c^	Present study

^a^Extrapolated from tumor xenograft mice

^b^Obtained by using female rat data

^c^Obtained by using male rat data

The radiation dose resulting from the i.v. injection of ^68^Ga-citrate has been estimated from ^67^Ga-citrate data [16]. The estimated absorbed dose for total body, calculated assuming a uniform distribution of radioactivity, was 0.052 rads/mCi. For ^68^Ge/^68^Ga generator eluate, the total-body radiation dose estimates for female and male rats were 0.02560 and 0.01730 mSv/MBq, respectively. However, the ^68^Ga-citrate and ^68^Ga-eluate values are not directly comparable because they are expressed with different units (rads/mCi vs. mSv/MBq), but assuming that only gamma radiation is taken into account for the energy dose, the rad value can be converted into the equivalent dose value rem, thus giving an absorbed dose of 0.052 rem/mCi = 0.014 mSv/MBq. There are limited number of studies comparing the PET tracer dosimetry in animals and humans. Table 8 contains a list of reference studies in which human effective doses derived from preclinical studies are reported and compared to effective doses from human measurements [17–24]. The absorbed doses and effective doses from the *in vivo* studies in rats may be different from those obtained in human studies because of the dissimilar physiology of rodents and humans. For example, the blood flow rate has a remarkable influence on the time-activity curve shape, the area under the curve, and the measured number of disintegrations.

**Table 8:** Comparison of preclinical studies with the human effective dose (ED) estimates

PET tracer	Animal derived ED (mSv/MBq)	Human derived ED (mSv/MBq)	Reference
^11^C-6-OH-BTA-1	0.0065^a^	0.0045	Parsey 2005; Scheinin 2007 [17, 18]
^11^C-MPGA	0.0048	0.0053	Santens 1998 [19]
6-^18^ F-Fluoro-L-Dopa	0.0539^b^	0.0199	Harvey 1985; Brown 1998 [20, 21]
^18^ F-FET	0.0186	0.0165	Tang 2003; Pauleit 2003 [22, 23]
^11^C-Choline	0.0028	0.0044	Tolvanen 2010 [24]

^a^Males only

^b^ED value calculated with the biological risk weight factors in accordance with ICRP 30 publication

In this study, scaling between rat and human data was performed using the overall non-organ-specific weight. In general, interspecies extrapolation of biokinetic data is based on the fact that the cellular structures and biochemistry are remarkably alike across the entire animal kingdom. Despite these similarities, however, the extrapolation of biokinetic data from laboratory animals to humans entails uncertainty, particularly for the liver, due to the qualitative differences among various species in the handling of many elements by this organ. Allometric scaling from laboratory animals to humans on the basis of body weight or surface area is the most commonly used method. It is based on the assumption that the biokinetics of compounds primarily depends on the metabolic rate of the animal and that the metabolic rate is a function of the body weight or body surface area of the animal. Yet, several other scaling methods have been proposed [25], based on, for example, the modeling of pharmacokinetic parameters where the variation of serum protein binding between species is taken into account.

## Conclusions

The estimated human radiation burden of the ^68^Ge/^68^Ga generator eluate was slightly higher for females and similar for males as compared with somatostatin ligands ^68^Ga-DOTANOC,^68^Ga-DOTATOC, and ^68^Ga-DOTATATE, which is probably due to the retention in the liver and spleen. Our results revealed some differences between female and male rat data, which, at least in part, may be explained by the small sample size.
