# Erratum to: Absorption, distribution and excretion of intravenously injected ^68^Ge/^68^Ga generator eluate in healthy rats, and estimation of human radiation dosimetry

**DOI:** 10.1186/s13550-016-0205-8

**Published:** 2016-06-13

**Authors:** Anu Autio, Helena Virtanen, Tuula Tolvanen, Heidi Liljenbäck, Vesa Oikonen, Tiina Saanijoki, Riikka Siitonen, Meeri Käkelä, Andrea Schüssele, Mika Teräs, Anne Roivainen

**Affiliations:** Turku PET Centre, Turku University Hospital and University of Turku, Turku, FI-20521 Finland; Turku Center for Disease Modeling, University of Turku, Turku, Finland; Eckert & Ziegler Radiopharma GmbH, Berlin, Germany

## Erratum

After publishing of the paper [1], substantial errors in Tables 4, 5, 6 and 7 have been noticed. The correct Tables 4, 5, 6 and 7 are presented here. Authors regret any inconvenience.

**Table 4:** Human residence times and radiation dose estimates for ^68^Ga radioactivity extrapolated from the rat biodistribution data

Organ	Residence time (h)		Dose (mSv/MBq)	
	Female rat data	Male rat data	Female rat data	Male rat data
Adrenals			0.01140	0.00931
Blood	0.2080	0.5000		
Bone	0.6645	0.3089		
Bone marrow	0.0907	0.0479		
Brain	0.0348	0.0326	0.01800	0.01340
Breasts			0.00587	0.00615
Gallbladder wall			0.00955	0.00810
Heart wall	0.0208	0.0187	0.17400	0 30300
Kidneys	0.0196	0.0100	0.03850	0.01970
Liver	0.2526	0.2653	0.09720	0.07660
Lower large intestine wall			0.00319	0.00152
Lungs	0.3174	0.2770	0.18600	0.13400
Muscle	0.1014	0.1084	0.00729	0.00510
Osteogenic cells			0.11600	0.04310
Ovaries	0.0004		0.01880	0.00169
Pancreas	0.0019	0.0023	0.01870	0.01870
Red marrow			0.02250	0.01380
Skin			0.000292	0.00202
Small intestine			0.00388	0.00222
Spleen			0.00554	0.00412
Stomach wall			0.0565	0.00477
Testes		0.000028		0.00077
Thymus			0.01000	0.00139
Thyroid	0.0087	0.0095	0.22100	0.19800
Upper large intestine wall			0.00399	0.00268
Urinary bladder wall			0.00232	0.00106
Uterus	0.0137		0.007920	0.00142
Whole body			0.01770	0.01150
Effective dose			0.0483	0.0338

**Table 5:** Additional dosimetry estimates based on a female rat distribution data

	Absorbed dose per unit radioactivity administered (mSv/MBq)				
Organ	15-year-old (50 kg)	10-year-old (30 kg)	5-year-old (17 kg)	1-year-old (10 kg)	Newborn (5 kg)
Adrenals	0.01120	0.01640	0.02380	0.04030	0.07820
Brain	0.01590	0.01760	0.02060	0.02920	0.06670
Breasts	0.00581	0.01100	0.01630	0.02690	0.05450
Gallbladder wall	0.00920	0.01270	0.02010	0.03900	0.07500
Heart wall	0.19400	0.30100	0.48300	0.87300	1.72000
Kidneys	0.04210	0.06000	0.08880	0.16000	0.41500
Liver	0.09740	0.14800	0.22000	0.42700	0.98900
Lower large intestine wall	0.00321	0.00504	0.00771	0.01330	0.02920
Lungs	0.22400	0.31900	0.49300	0.98400	2.71000
Muscle	0.00757	0.01310	0.03190	0.06220	0.09540
Osteogenic cells	0.11400	0.18400	0.31000	0.73500	2.35000
Ovaries	0.02030	0.05660	0.09880	0.22500	0.45900
Pancreas	0.002180	0.04060	0.05470	0.11200	0.34000
Red marrow	0.02560	0.04150	0.07770	0.17700	0.57100
Skin	0.00281	0.00435	0.00671	0.01220	0.02710
Small intestine	0.00386	0.00619	0.00986	0.01780	0.03760
Spleen	0.00562	0.00858	0.01300	0.02380	0.04920
Stomach wall	0.00559	0.00800	0.01330	0.02500	0.05020
Thymus	0.01020	0.01330	0.01900	0.02970	0.05700
Thyroid	0.29800	0.46000	1.02000	1.93000	2.63000
Upper large intestine wall	0.00389	0.00669	0.01040	0.01900	0.04250
Urinary bladder wall	0.00223	0.00377	0.00625	0.01100	0.02220
Uterus	0.08020	1.34000	2.03000	3.69000	1.47000
Total body	0.01780	0.02890	0.04680	0.09200	0.23400
Effective dose (mSv/MBq)	0.05740	0.12300	0.20900	0.41000	0.71700

**Table 6:** Additional dosimetry estimates based on a male rat distribution data

	Absorbed dose per unit radioactivity administered (mSv/MBq)				
Organ	15-year-old (50 kg)	10-year-old (30 kg)	5-year-old (17 kg)	1-year-old (10 kg)	Newborn (5 kg)
Adrenals	0.01120	0.01650	0.02350	0.03770	0.07490
Brain	0.01370	0.01480	0.01700	0.02410	0.05630
Breasts	0.00743	0.01420	0.02130	0.03500	0.07250
Gallbladder wall	0.00960	0.01370	0.02130	0.04090	0.08030
Heart wall	0.39300	0.61100	0.98300	1.78000	3.4900
Kidneys	0.02410	0.03450	0.05100	0.09110	0.23100
Liver	0.10300	0.15700	0.23300	0.45000	1.04000
Lower large intestine wall	0.00197	0.00308	0.00506	0.00907	0.02040
Lungs	0.20000	0.28500	0.43900	0.87200	2.38000
Muscle	0.00739	0.01290	0.03260	0.06360	0.09610
Pancreas	0.02570	0.04800	0.06460	0.13100	0.40300
Red marrow	0.01540	0.02430	0.04410	0.09800	0.31100
Osteogenic cells	0.05580	0.09010	0.15100	0.35600	1.13000
Skin	0.00244	0.00362	0.00572	0.01030	0.02320
Small intestine	0.00287	0.00483	0.00796	0.01460	0.03090
Spleen	0.0327	0.0503	0.0801	0.1460	0.3780
Stomach wall	0.000657	0.00988	0.01530	0.02870	0.05600
Testes	0.00178	0.00745	0.00935	0.01380	0.02390
Thymus	0.01580	0.01940	0.02760	0.04170	0.07940
Thyroid	0.32500	0.50200	1.12000	2.1100	2.88000
Upper large intestine wall	0.00327	0.00581	0.00935	0.01820	0.03850
Urinary bladder wall	0.00129	0.00224	0.00393	0.00699	0.01520
Total body	0.01470	0.02370	0.03830	0.07480	0.19000
Effective dose (mSv/MBq)	0.05060	0.07560	0.13400	0.26000	0.55500

**Table 7:** Human effective doses of ^68^Ga radiopharmaceuticals and ^18^F-FDG

Radiopharmaceutical	Effective dose (mSv/MBq)	Reference
^68^Ga-DOTANOC	0.025	Pettinato 2008 [10]
^68^Ga-DOTATOC	0.023	Hartmann 2009 [11]
^68^Ga-DOTATATE	0.021	Sandström 2013 [12]
^68^Ga-NOTA-2Rs15d	0.0218^a^	Xavier 2013 [13]
BAY86-7548	0.051	Roivainen 2013 [14]
^18^F-FDG	0.0190	ICRP Publication 1998 [15]
^68^Ga-eluate	0.0483^b^, 0.0338^c^	Present study

^a^Extrapolated from tumour xenograft mice; ^b^Obtained by using female rat data; ^c^Obtained by using male rat data
